# Long-term trends of lung cancer incidence and survival in southeastern China, 2011–2020: a population-based study

**DOI:** 10.1186/s12890-024-02841-0

**Published:** 2024-01-10

**Authors:** Yan Zhou, Zhisheng Xiang, Weikai Lin, Jinghui Lin, Yeying Wen, Linrong Wu, Jingyu Ma, Chuanben Chen

**Affiliations:** 1 Department of Epidemiology, Clinical Oncology School of Fujian Medical University, Fujian Cancer Hospital, 350014 Fuzhou, China; 2 Fujian Key Laboratory of Advanced Technology for Cancer Screening and Early Diagnosis, 350014 Fuzhou, China; 3 Department of Thoracic Surgery, The Second Affiliated Hospital of Fujian University of Traditional Chinese Medicine, 350003 Fuzhou, China; 4 Department of Thoracic oncology, Clinical Oncology School of Fujian Medical University, Fujian Cancer Hospital, 350014 Fuzhou, China; 5 Fujian Provincial Office for Cancer Prevention and Control, 350014 Fuzhou, China; 6 Department of Radiation Oncology, Clinical Oncology School of Fujian Medical University, Fujian Cancer Hospital, No.420 Fuma Road, 350014 Fuzhou, China

**Keywords:** Lung cancer, Incidence, Survival, Trend, Population-based study

## Abstract

**Background:**

Lung cancer is the primary cause of cancer-related deaths in China. This study analysed the incidence and survival trends of lung cancer from 2011 to 2020 in Fujian Province, southeast of China, and provided basis for formulating prevention and treatment strategies.

**Methods:**

The population-based cancer data was used to analyse the incidence of lung cancer between 2011 and 2020, which were stratified by sex, age and histology. The change of incidence trend was analysed using Joinpoint regression. The relative survival of lung cancer with onset in 2011–2014, 2015–2017 and 2018–2020 were calculated using the cohort, complete and period methods, respectively.

**Results:**

There were 23,043 patients diagnosed with lung cancer in seven registries between 2011 and 2020, with an age-standardized incidence rate (ASIR) of 37.7/100,000. The males ASIR increased from 51.1/100,000 to 60.5/100,000 with an annual percentage change (APC) of 1.5%. However, females ASIR increased faster than males, with an APC of 5.7% in 2011–2017 and 21.0% in 2017–2020. Compared with 2011, the average onset age of males and females in 2020 was 1.5 years and 5.9 years earlier, respectively. Moreover, the proportion of adenocarcinoma has increased, while squamous cell carcinoma and small cell carcinoma have decreased over the past decade. The 5-year relative survival of lung cancer increased from 13.8 to 23.7%, with a greater average increase in females than males (8.7% and 2.6%). The 5-year relative survival of adenocarcinoma, squamous cell carcinoma and small cell carcinoma reached 47.1%, 18.3% and 6.9% in 2018–2020, respectively.

**Conclusions:**

The incidence of lung cancer in Fujian Province is on the rise, with a significant rise in adenocarcinoma, a younger age of onset and the possibility of overdiagnosis. Thus, Fujian Province should strengthen the prevention and control of lung cancer, giving more attention to the prevention and treatment of lung cancer in females and young populations.

**Supplementary Information:**

The online version contains supplementary material available at 10.1186/s12890-024-02841-0.

## Background

According to the GLOBOCAN 2020 estimates report that lung cancer has the second highest incidence worldwide (2,210,000 new cases or 11.4% of total cancer incidence in 2020) and is the primary cause of cancer-related deaths (1,800,000 cases or 18.0%) [1]. The incidence of lung cancer in females is rising in most countries, while that in males is declining [2]. The 5-year survival for lung cancer has increased in recent years but it is much lower than other major cancers, such as breast cancer or colon cancer [3]. These changes in trends can be mainly attributed to the control of tobacco use, the prevalence of computed tomography (CT) screening and the use of new therapeutic agents [4–7]. According to the National Cancer Center of China, approximately 828,100 new cases and 657,000 lung cancer-related deaths were reported in China in 2016, with the number of new cases and deaths increasing by 162.6% and 123.6% respectively between 2000 and 2016. This increase has been speculated to be mainly due to ageing. In males, the age-standardized incidence rate increased by approximately 0.8% per year, and the annualized growth rate in females reached 2.1% [8]. The 5-year relative survival of lung cancer in China increased from 16.1 to 19.7% between 2003 and 2015 [9]. However, long-term epidemiological studies of lung cancer in the local areas of China are scarce. Therefore, in this study, changes in lung cancer incidence trends and survival were analysed using the data of cancer registry and death surveillance from 2011 to 2020 in Fujian Province, Southeastern China. Moreover, the conclusions may provide important clues for lung cancer prevention and treatment in this region.

## Methods

### Data collection and quality control

The Fujian Cancer Prevention and Control Office is responsible for population-based cancer surveillance and statistics in the province and has been conducting population-based cancer registries since 2009. In this study, we obtained the incidence and follow-up data of patients with lung cancer diagnosed between January 1, 2011, and December 31, 2020, from seven cancer registries in Fujian Province, covering a population of 5,192,272 in 2020 (2,594,514 males and 2,597,758 females).

The registry collected information on new cancer cases from hospitals in its jurisdiction and uses a combination of passive and active follow-up methods to obtain patient survival status. The registry regularly obtained population-based cause-of-death surveillance data, matched it with cancer incidence data, the cases matched successfully was supplemented the time and underlying cause of death, which was known as passive follow-up. Active follow-up involved the collection of the survival status of cases not matched with the cause of death data in the incidence data through telephone and physical visit. All cases were followed up for survival outcome status until December 31, 2021. Demographic information for each registry was obtained from the Public Security Household Registration Department. The registries used the 10th edition of the International Classification of Diseases (ICD-10) and the 3rd edition of the International Classification of Diseases for Oncology (ICD-O-3) to code cancer cases. The cases with ICD-10 codes C33-C34 were included in this study. Additionally, the histological subtypes of lung cancer were classified into small cell carcinoma, adenocarcinoma, squamous carcinoma, large cell carcinoma and other subtypes according to the 2004 World Health Organization (WHO) cancer classification criteria. The Ethics Committee of Fujian Cancer Hospital determined that ethics approval and informed consent were not required for this research, because this is a retrospective study that used source data that were completely unidentifiable, and all data were anonymous. The research was conducted in accordance with the Declaration of Helsinki, and the research data were kept confidential.

All submitted data underwent quality control analysis following the Chinese Guideline for Cancer Registration (version 2016) and data review rules established by the International Agency for Research on Cancer/International Association of Cancer Registries [10–12]. Cases that did not meet the quality requirements were returned to the local registry to verify the authenticity of the data. The result of quality control index of lung cancer in Fujian Province from 2011 to 2020 was shown in Additional file 1. In the survival analysis, cases based only on death certificates or cases where survival outcomes could not be determined, or cases with more than two primary malignant tumours were excluded.

### Statistical analyses

The crude incidence rate, the age-standardized rates (ASR) and age-specific incidence rates were calculated using the SAS 9.0. The age-standardized incidence rates (ASIR) were calculated using Segi^’^s population composition [13]. The long-term trends of the incidence were estimated using Joinpoint Regression Models, with calculating the annual percentage change (APC), average annual percentage change (AAPC) and 95% confidence interval (CI). As relevant histological information was not reported for some cases, the incidence of histological type of lung cancer was estimated by the product of the histological type composition ratio and the total incidence rate for each year. The relative survival was calculated using the Strs package of the Stata 15.1, which calculated the ratio of the observed survival rate to the population’s expected survival rate. The observed and expected survival rates were calculated using the life table method and Ederer II model, respectively. The year of diagnosis was divided into three different periods (2011–2014, 2015–2017 and 2018–2020) for the analysis of survival, and the relative survival for the three periods were calculated using the cohort, complete and period methods, respectively [14]. The weighted least squares regression was used to analyse the change of relative survival in different calendar periods. The variance of each survival estimate was used as a weight of linear regression. The slope of linear regression provides an estimate of the average change between successive periods of diagnosis, and 95% CI comes from its standard error [9]. Patients were divided into five main age groups (0–44, 45–54, 55–64, 65–74 and ≥ 75 years) to analyse the temporal changes in incidence and relative survival in different age groups. Statistical significance was set at α < 0.05.

## Results

### Overall lung cancer incidence

A total of 23,043 patients were diagnosed with lung cancer during 2011–2020 (15,450 males and 7,593 females), with a mean patient age of onset at 64.9 ± 12.1 (65.6 ± 11.2 years for males and 63.4 ± 13.5 years for females). A total crude incidence rate was 49.5/100,000 in 2011 to 2020 (66.0/100,000 for males and 32.9/100,000 for females), and ASIR was 37.7/100,000 in total (53.7/100,000 for males and 23.6/100,000 for females) and a cumulative incidence rate (0–74 years) was 4.7% (6.7% for males and 2.8% for females).

### Time trends in lung cancer incidence

**Fig. 1:**
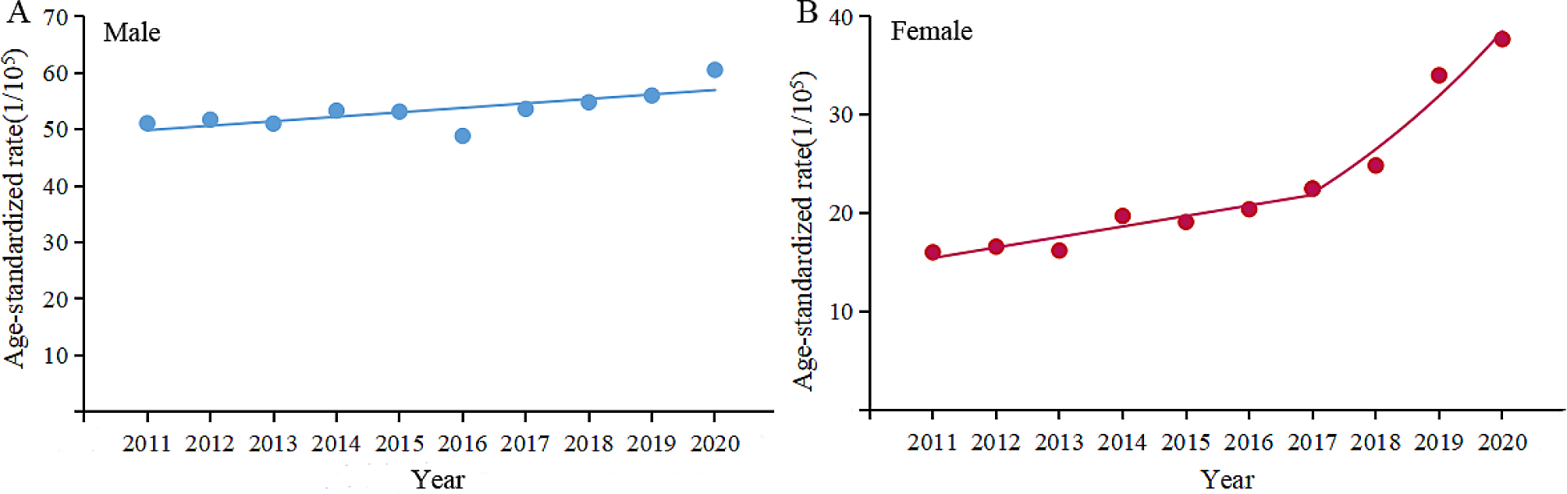
The trend of age-standardized incidence rate of lung in males (A) and females (B) between 2011 and 2020

Overall, lung cancer ASIR increased from 32.8/100,000 to 48.3/100,000 during 2011–2020, revealing an increase of 47.3%. Males ASIR increased from 51.1/100,000 to 60.5/100,000, with an APC of 1.5% (95%CI: 0.4–2.6). However, females ASIR increased faster than males, from 16.0/100,000 to 37.7/100,000, with an APC of 5.7% (95%CI: 1.8–9.7) in 2011–2017 and 21.0% (95%CI: 8.4–35.1) in 2017–2020. Moreover, the ratio of males to females ASIR decreased from 3.2 in 2011 to 1.6 in 2020 (Table 1; Fig. 1).

**Table 1:** The incidence of lung cancer in Fujian Province from 2011 to 2020

Year		Total				Male				Female		
	Population	Case	Crude incidence(1/10^5^)	ASIR(1/10^5^)	Population	Case	Crude incidence(1/10^5^)	ASIR(1/10^5^)	Population	Case	Crude incidence(1/10^5^)	ASIR(1/10^5^)
2011	4204105	1608	38.2	32.8	2123361	1186	55.9	51.1	2080744	422	20.3	16.0
2012	4275298	1727	40.4	33.1	2158437	1266	58.7	51.7	2116861	461	21.8	16.6
2013	4403069	1833	41.6	32.7	2221040	1328	59.8	51.0	2182029	505	23.1	16.2
2014	4469340	2009	45.0	35.7	2255015	1403	62.2	53.3	2214325	606	27.4	19.7
2015	4579663	2149	46.9	35.1	2305769	1524	66.1	53.2	2273894	625	27.5	19.1
2016	4659016	2170	46.6	34.0	2345274	1489	63.5	48.8	2313742	681	29.4	20.4
2017	4751884	2328	49.0	36.9	2393297	1572	65.7	53.6	2358587	756	32.1	22.5
2018	4944103	2649	53.6	38.9	2482433	1759	70.9	54.8	2461670	890	36.2	24.8
2019	5024895	3087	61.4	44.2	2517817	1850	73.5	56.0	2507078	1237	49.3	34.0
2020	5192272	3483	67.1	48.3	2594514	2073	79.9	60.5	2597758	1410	54.3	37.7

ASIR, age-standardized incidence rates

### Changes in lung cancer incidence in different age groups

**Fig. 2:**
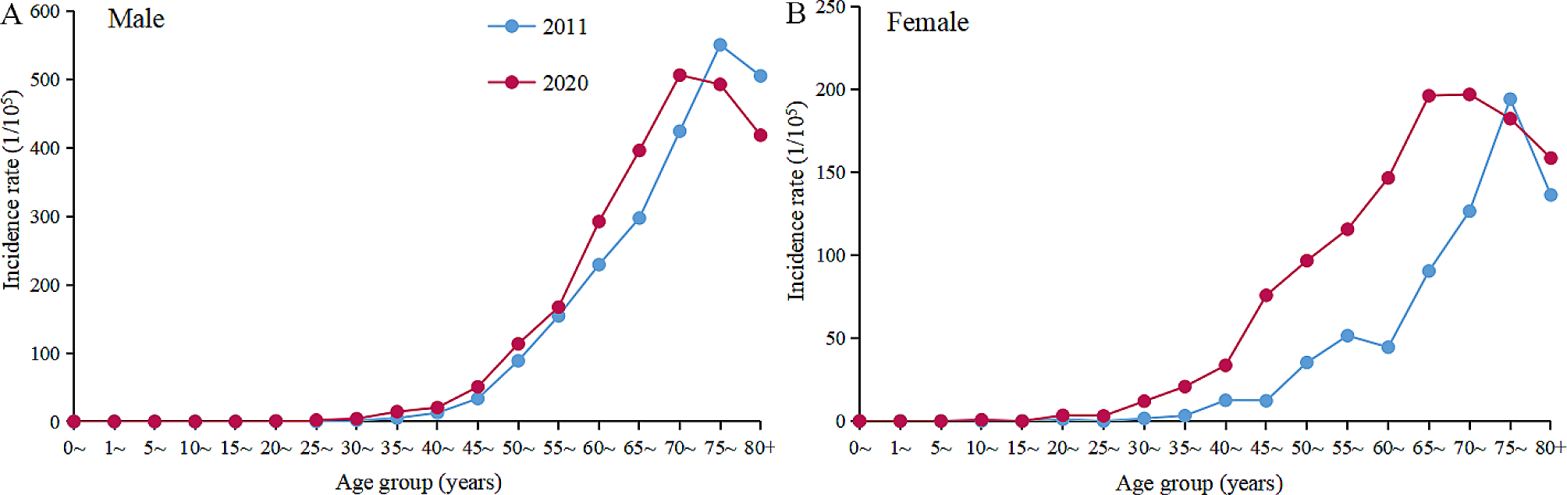
The age-specific incidence of lung cancer in males **(A)** and females **(B)**

An increasing trend in the incidence in the age groups < 45 years, 45–54 years, 55–64 years and 65–74 years in males and females during 2011–2020 was observed, with males AAPC was 7.0%(95%CI: 1.1–13.3), 3.0%(95%CI: 0.2–6.0), 1.7%(95%CI: 0.3–3.2) and 1.9%(95%CI: 0.3–3.2), respectively. The AAPC of females was 20.8%(95%CI: 14.1–27.8), 14.5%(95%CI: 10.0–19.2), 11.0%(95%CI: 7.4–14.8) and 7.6%(95%CI: 4.6–10.6), respectively. The incidence rate of females in all age groups increasing faster than males. Notably, the incidence rate of females in the < 45 and 45–54 age groups reached 10.1/100,000 and 85.5/100,000 in 2020, respectively, which already exceeded the incidence rate of males in the same age groups (5.3/100,000 and 79.7/100,000, respectively). However, there was no significant change in the incidence rates for males and females in the ≥ 75 years group (Table 2). The mean age of lung cancer incidence in 2020 was 64.5 ± 11.1 years and 60.0 ± 13.2 years for males and females, respectively. From 2011 to 2020, the onset age of males and females was 1.5 years and 5.9 years earlier, respectively (Fig. 2).

**Table 2:** Trend of lung cancer incidence in different age groups from 2011 to 2020

Sex	Age group	Incidence(1/10^5^)		AAPC(%)	(95%CI)	*P*-value
		2011	2020			
Male	0–44	2.2	5.3	7.0	1.1–13.3	< 0.05
	45–54	56.4	79.7	3.0	0.2-6.0	< 0.05
	55–64	186.9	224.1	1.7	0.3–3.2	< 0.05
	65–74	352.0	439.6	1.9	0.7–3.2	< 0.05
	≥ 75	529.7	453.7	-1.3	-2.7-0.1	> 0.05
Female	0–44	2.2	10.1	20.8	14.1–27.8	< 0.05
	45–54	21.8	85.5	14.5	10.0-19.2	< 0.05
	55–64	48.5	130.0	11.0	7.4–14.8	< 0.05
	65–74	106.4	196.5	7.6	4.6–10.6	< 0.05
	≥ 75	163.0	168.2	1.3	-1.3-3.9	> 0.05
Total	0–44	2.2	7.6	13.9	8.4–19.4	< 0.05
	45–54	39.3	82.6	7.4	3.9–11.0	< 0.05
	55–64	116.9	175.6	4.3	2.4–6.2	< 0.05
	65–74	229.5	310.9	3.2	1.7–4.6	< 0.05
	≥ 75	313.1	288.4	-0.1	-1.4-1.2	> 0.05

AAPC, average annual percentage change; CI, confidence interval

### Changes of incidence in different histological types

**Fig. 3:**
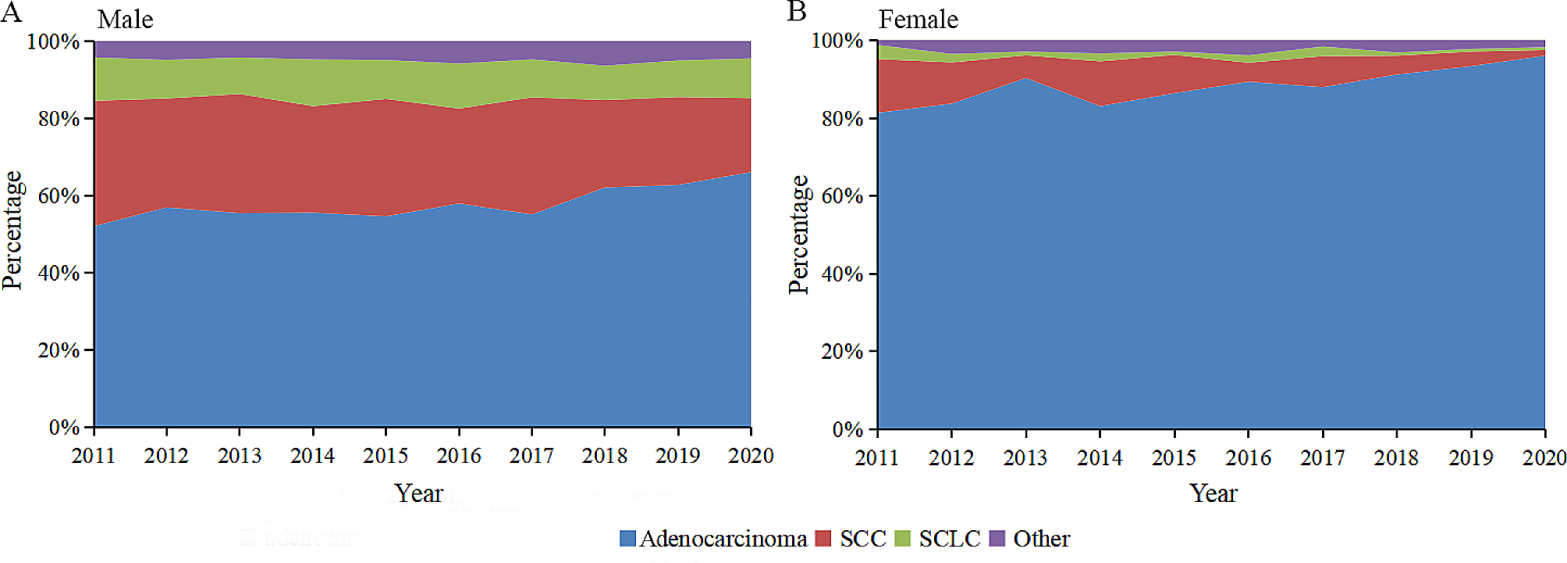
The proportion of lung cancer with different histological classification in males **(A)** and females **(B)** from 2011 to 2020. SCC, squamous cell carcinoma; SCLC, small cell lung carcinoma

A total of 13,567 cases had detailed histological classification, accounting for 58.9% of all cases. The proportions of histological diagnosis in the age groups < 45, 45–54, 55–64, 65–74 and ≥ 75 years were 77.4%, 71.2%, 65.5%, 60.0% and 37.3%, respectively. The common cases with the histological diagnosis were adenocarcinoma (9,593 cases, 70.7%), squamous carcinoma (2,481 cases, 18.3%) and small cell carcinoma (771 cases, 5.7%). Additionally, with a smaller number of other histological types (549 cases, 4.0%) included large cell carcinoma. As seen in Fig. 3, between 2011 and 2020, the proportion of adenocarcinoma in males and females increased while that of squamous carcinoma decreased. The proportion of small cell carcinoma remained the same in males and decreased in females, whereas that of other histological types showed no significant change.

**Fig. 4:**
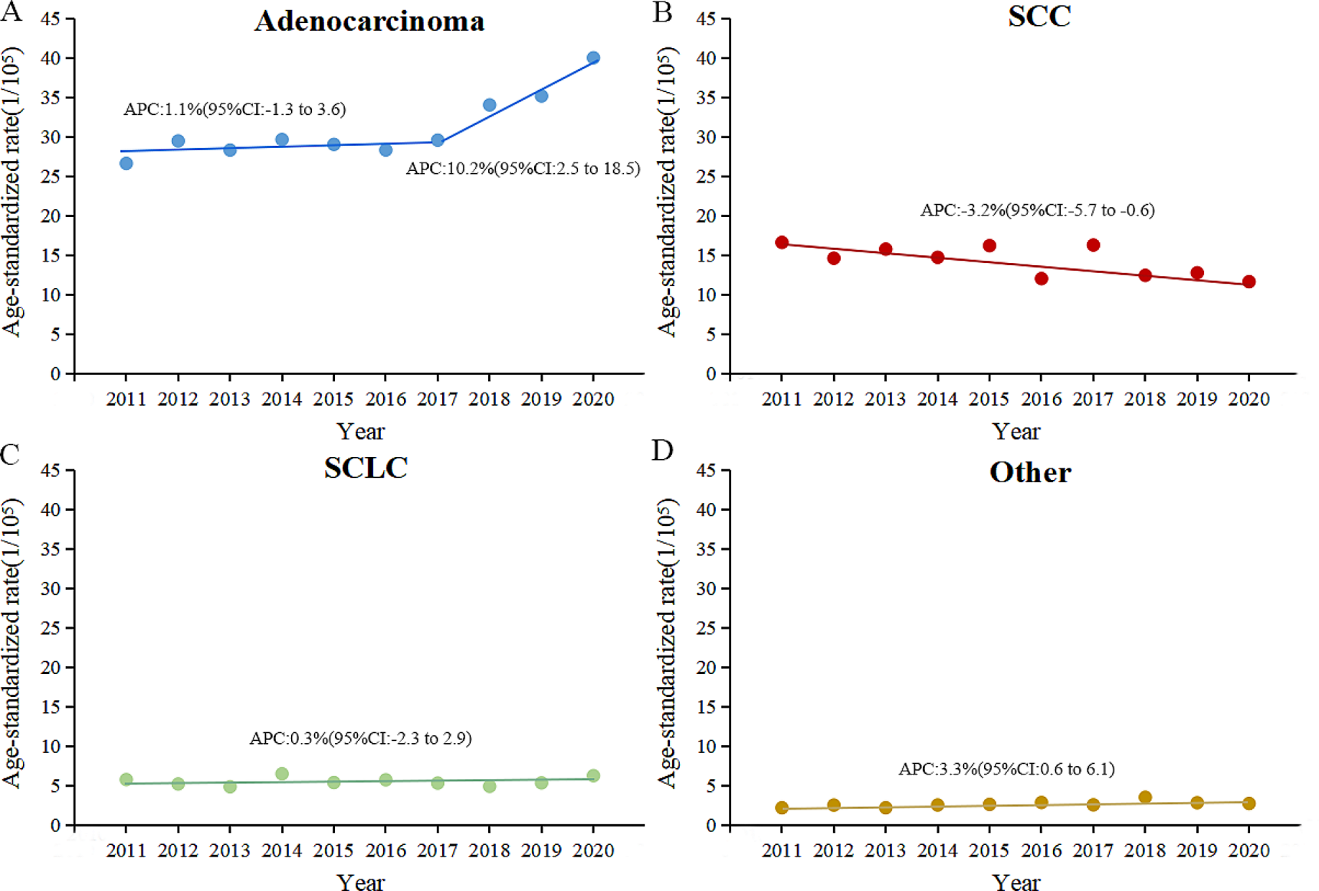
The trend of age-standardized incidence rate of lung adenocarcinoma **(A)**, SCC **(B)**, SCLC **(C)** and other histological classification of lung **(D)** in males from 2011 to 2020. SCC, squamous cell carcinoma; SCLC, small cell lung carcinoma; APC, annual percentage change; CI, confidence interval

**Fig. 5:**
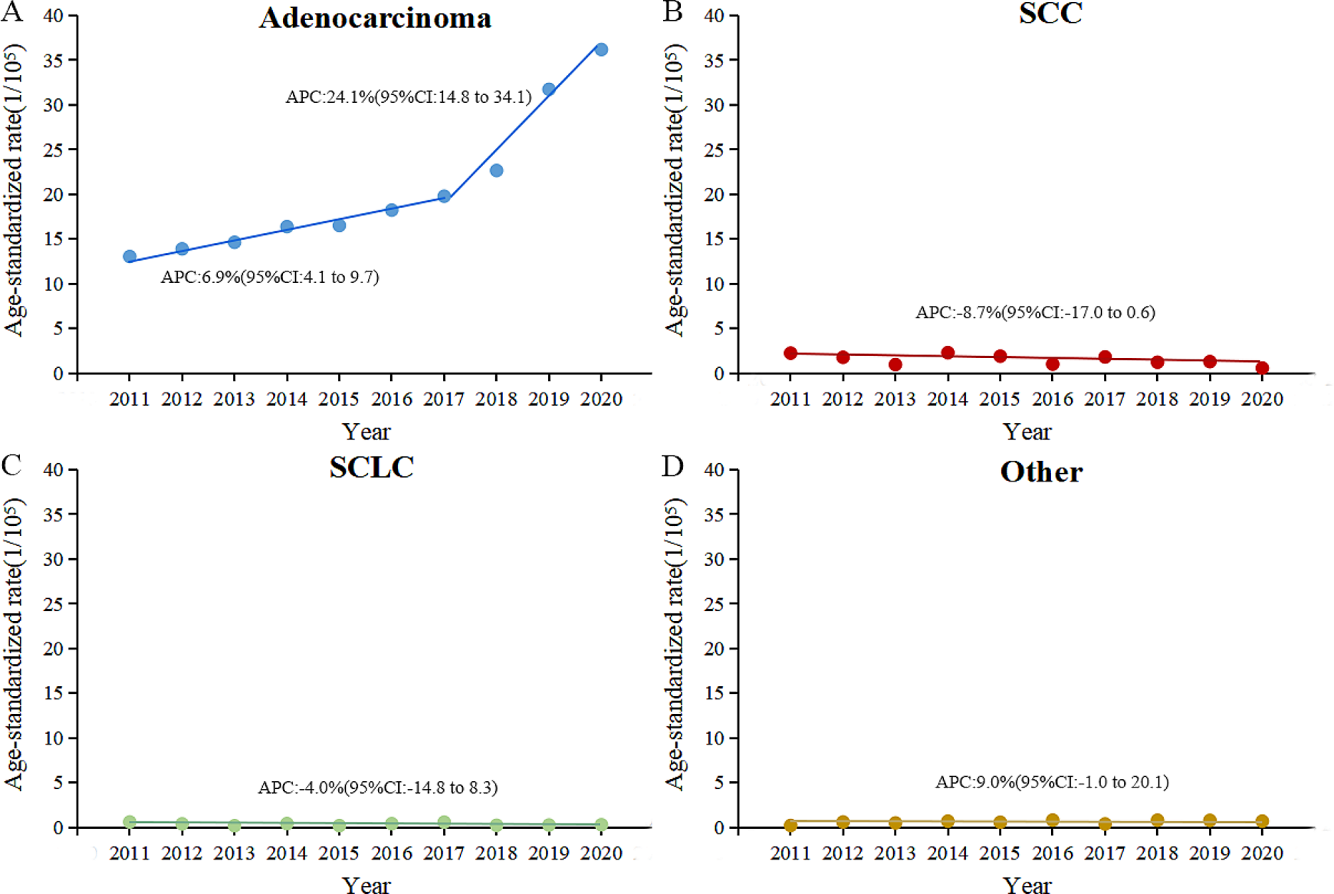
The trend of age-standardized incidence rate of lung adenocarcinoma **(A)**, SCC **(B)**, SCLC **(C)** and other histological classification of lung **(D)** in females from 2011 to 2020. SCC, squamous cell carcinoma; SCLC, small cell lung carcinoma; APC, annual percentage change; CI, confidence interval

On estimating incidence rates by the percentage of histological type by each year, adenocarcinoma in males increased by 50.3% (26.6/100,000 to 40.0/100,000) from 2011 to 2020, with a significant increase from 2017 to 2020 and an APC of 10.2%(95%CI: 2.5–18.5). Squamous carcinoma decreased by 30.0% (16.6/100,000 to 11.6/100,000), with an APC of -3.2%(95%CI: -5.7–-0.6). Notably, no significant change was observed in the small cell carcinoma incidence trend. Adenocarcinoma in females increased by 177.7% (13.0/100,000 to 36.2/100,000), with an APC of 6.9%(95%CI: 4.1–9.7) from 2011 to 2017, and an accelerated rise from 2017 to 2020 with an APC of 24.1%(95%CI: 14.8–34.1). Additionally, there was no downtrend of ASIR of squamous cell carcinoma (APC: -8.7%,95%CI: -17.0–0.6) and small cell carcinoma (APC: -4.0%,95%CI: -14.8–8.3) in females from 2011 to 2020 (Figs. 4 and 5).

### Analysis of the relative survival of lung cancer

The 5-year relative survival of patients with lung cancer increased from 13.8% (95%CI: 12.9–14.7) in 2011–2014 to 23.7% (95%CI: 22.4–24.9) in 2018–2020, with average change of 4.9%(95%CI:0.8-9.0). The 5-year relative survival increased in the 45–54 and 55–64 age groups, respectively, reaching 38.6% (95%CI: 34.7–42.5) and 29.0% ( 95%CI: 26.7–31.4). However, there was no average change of 5-year relative survival in the < 45, 65–74 and ≥ 75 age group. The 5-year relative survival of adenocarcinoma improved from 20.6% (95%CI: 18.6–22.4) to 47.1% (95%CI: 44.6–49.6), exhibiting a substantial increase of 13.2%(95%CI:9.7–16.7). Furthermore, the survival rate of squamous carcinoma and small cell carcinoma was not changed from 2011 to 2020, with average change of 0.9% (95%CI:-11.2-13.1) and 0.1% (95%CI:-10.8-11.1) (Table 3).

**Table 3:** The 5-year relative survival of lung cancer in different time periods

Characteristic	Case	2011–2014			2015–2017			2018–2020		Average change(95%CI)
		5-year RS(%)	95%CI		5-year RS(%)	95%CI		5-year RS(%)	95%CI	
Total	22,153	13.8	12.9–14.7		18.2	17.2–19.3		23.7	22.4–24.9	4.9%(0.8-9.0)
Sex										
Male	8336	11.9	11.0-12.9		14.5	13.4–15.7		17.2	15.9–18.5	2.6%(2.3-3.0)
Female	4660	18.4	16.6–20.4		26.3	24.3–28.5		36.0	33.4–38.6	8.7%(1.9–15.5)
Age group										
<45	1129	19.9	15.4–24.9		40.1	33.9–46.2		48.4	41.1–55.4	15.0%(-26.9-56.9)
45–54	3110	15.5	13.1–18.1		27.7	24.6–30.8		38.6	34.7–42.5	11.6%(6.9–16.4)
55–64	6280	15.7	14.1–17.4		23.0	21.0-25.1		29.0	26.7–31.4	6.7%(1.9–11.5)
65–74	6489	13.2	11.7–14.9		14.8	13.1–16.7		19.4	17.4–21.5	2.9%(-7.9-13.9)
≥ 75	5145	9.8	8.2–11.6		7.9	6.4–9.7		7.9	6.1–10.1	-1.0%(-8.3-6.3)
Histology Type										
Adenocarcinoma	9158	20.6	18.6–22.4		33.4	31.3–35.4		47.1	44.6–49.6	13.2%(9.7–16.7)
Squamous cell carcinoma	2383	16.3	13.6–19.3		15.7	13.0-18.6		18.3	15.3–21.5	0.9%(-11.2-13.1)
Small cell carcinoma	927	7.0	4.2–10.8		5.4	3.0-8.8		6.9	4.2–10.5	0.1%(-10.8-11.1)
Other	528	18.7	12.1–26.5		23.0	16.3–30.5		21.4	15.3–28.3	1.3%(-19.9-23.4)

RS, relative survival; CI, confidence interval

## Discussion

Fujian Province is located in Southeastern China, across the sea from Taiwan, and is the fourth wealthiest province in China, with a per capita GDP of approximately US$15,000 in 2020. Fujian has a forest coverage rate of 66.8%, ranking first in the country in terms of air quality [15]. This study is the first comprehensive analysis of lung cancer incidence trends and survival in Fujian Province based on surveillance data from the seven earliest cancer registries in Fujian Province. The ASIR of lung cancer was 37.7/100,000 in Fujian Province from 2011 to 2020, which is higher than the global average (22.4/100,000) and eastern China (36.4/100,000 in 2016) [2, 8]. The ASIR in males showed a slowly increasing trend with an APC of 1.5%(95%CI: 0.4–2.6). While incidence of females increased substantially faster than males, with an APC of 5.7%(95%CI: 1.8–9.7) in 2011–2017 and 21.0%(95%CI: 8.4–35.1) in 2017–2020. This incidence trend of lung cancer was observed to be similar to that of Shanghai [2, 16].

We analysed the change in the age of onset, and found that compared with 2011, the mean age of onset of males and females in 2020 was 1.5 and 5.9 years earlier, respectively. Incidence increased significantly in all age groups except for the ≥ 75 years group, with females increasing faster than males, especially in the < 45 years and 45–54 years groups. Notably, females in these age groups surpassed males in incidence in 2020. We found that adenocarcinoma increased by 50.3% and 177.7% in males and females, respectively. However, squamous carcinoma showed a downward trend, and the incidence of small cell carcinoma remained unchanged. Thus, the above analysis revealing that the rising incidence of adenocarcinoma contributes to the increased lung cancer incidence in Fujian.

Smoking is highly associated with lung cancer incidence, and owing to tobacco control, most countries have shown a decreasing trend in males lung cancer [2, 17, 18]. The smoking rate in Fujian Province in 2016–2017 was 48.9% for males and only 0.9% for females, which is lower than the national average [19, 20]. With such a low prevalence of smoking among females, it is unlikely to be the main reason for their rising incidence of lung cancer. Recently, the Chinese government has taken strong tobacco control measures, thereby decreasing the smoking prevalence and intensity in the population since 2010 [20]. Moreover, a survey result showed that the proportion of second-hand smoke exposure among non-current smokers aged ≥ 15 years in China decreased in 2018 compared with 2010 [21]. These results highly showed the unlikeliness that smoke exposure under such a strict policy would lead to a rise in incidence of lung cancer in Fujian Province.

Air pollution is likely to contribute to the increased incidence of lung cancer, with some evidence supporting the fact that exposure to particulate matter (PM) 2.5 is associated with an increased risk of lung cancer incidence and mortality [22, 23]. A study have suggested that environmental PM pollution is the second most important factor after smoking in lung cancer deaths in China, accounting for 22.63% of the attributable risk of death [24]. Fujian Province has the highest forest cover in the country and better air quality. However, the impact of PM 2.5 on the incidence of lung cancer in Fujian Province have not been well evaluated and need further study. In addition to common risk factors, some studies have suggested that sex hormones may be associated with the development of non-small cell lung cancer in females [25, 26].

The most significant findings of the current study are the earlier age of lung cancer onset and a substantial increase in adenocarcinoma incidence (especially in females), which suggest that it may be related to the increased detection secondary to more widely adopted low-dose spiral CT (LDCT). Since the publication of the US Lung Screening Trial (NLST) in 2011, wherein LDCT screening was reported to have reduced lung cancer mortality by 20.0% in high-risk groups. LDCT screening has been widely used in China for employee health screening, whether or not the examinee is in a high-risk group for smoking [27]. LDCT is available at a low price in China, costing approximately US$30–40 for a single examination, which results to a high participation rate even among individuals without medical insurance coverage. Moreover, the data on lung cancer incidence in Shanghai showed similar results to this study, with a rapid increase in females between 2011 and 2017 (APC of 11.98%). Additionally, Shanghai also reports the highest increase in young females and an increasing trend in incidence mainly in early-stage cancer and lung adenocarcinoma. However, the incidence of late-stage cases did not decrease, suggesting that LDCT examinations may lead to the overdiagnosis of lung cancer in females [28]. A study in Taiwan also concluded that in mostly non-smoking Asian females, LDCT screening was associated with lung cancer overdiagnosis [29].

The 5-year relative survival of patients with lung cancer in Fujian Province increased from 13.8 to 23.7% in the decade, with females increasing greater than males (8.7% and 2.6%). The findings also suggested that the survival rate of younger patients is higher than that of the elderly. The relative survival for adenocarcinoma increased from 20.6 to 47.1%, with a significant increase of 13.2% in the period. However, no significant change was observed in survival rates of other types. Increased survival rates in lung cancer have been reported in many studies in recent years [4, 10, 30], which has been attributed mainly to the breakthroughs in targeted therapies and immunotherapy, as well as LDCT screening for better prognosis in early cases. EGFR mutations, ALK translocations, ROS1 aberrations and BRAF mutations are now routinely evaluated in advanced non-small cell lung cancer (NSCLC), especially in adenocarcinoma. And tyrosine kinase inhibitors (TKIs) have become the standard first-line treatment for patients with advanced EGFR, ALK or ROS1 mutation-positive NSCLC. Such breakthroughs in targeted therapies have improved survival and the quality of life in patients with NSCLC [7, 31]. Since 2015, the clinical practice of immunotherapy for advanced lung cancer has seen significant advances, especially PD-1/PD-L1 inhibitors have improved the survival outcomes in patients with NSCLC, with approximately 20% of patients having sustained responses [32, 33]. Furthermore, LDCT screening of high-risk populations facilitates early diagnosis and a greater likelihood of successful cure, and the effectiveness of LDCT screening in reducing lung cancer mortality has been demonstrated in several independent international randomised controlled clinical trials [4, 34–36]. In the high-risk Chinese population, the screening group that attended one LDCT scan had a 31.0% reduction in mortality compared to the non-screening group [37].

There are certain shortcomings in this study. There are no compulsive requirements for the collection of staging data for the cancer registry in Fujian Province, thus posing a challenge in understanding the magnitude of changes in the incidence of early and late stage lung cancer. A definitive judgment of overdiagnosis can’t be made at this time. The exact histological classification was not reported in some cases, which could be due to the lack of further treatment after imaging diagnosis in older cases. The incidence of each type of lung cancer can only be estimated based on the composition, which could have introduced bias. Nonetheless, based on the available basic information, we hypothesise that the significant increase in the incidence of lung adenocarcinoma in recent years cannot be fully explained by smoking or air pollution changes in risk exposure. Combined with the earlier age of onset, the possibility of overdiagnosis due to the widespread use of LDCT screening has also been speculated. Thus, with the increase in lung cancer detection rate in people, we should emphasize strengthening etiological studies [38].

## Conclusion

The incidence of lung cancer in Fujian Province is rising continuously, especially among young people. Although the survival rate has improved, the 5-year survival rate is still low. Therefore, it is necessary to strengthen the prevention and control of lung cancer. Moreover, it is an urgent need to enhance tobacco control in the population, improve the identification and early screening of high-risk groups, and at same time address the management of LDCT screening standards and screening nodules to avoid overtreatment.

### Electronic supplementary material

Below is the link to the electronic supplementary material.

Supplementary Material 1

## Data Availability

The datasets used and/or analysed during the current study are available from the corresponding author on reasonable request.

## Abbreviations

WHO: World Health Organization
ASR: Age-standardized rates
ASIR: Age-standardized incidence rate
APC: Annual percentage change
AAPC: Average annual percentage change
CI: Confidence interval
PM: Particulate matter
LDCT: Low-dose spiral computed tomography
