# Supplementary material for: Long-term trends of lung cancer incidence and survival in southeastern China, 2011–2020: a population-based study
Source: BMC Pulm Med. 2024 Jan 10;24:25. doi: 10.1186/s12890-024-02841-0 (PMC10782768; doi:10.1186/s12890-024-02841-0)
Supplement: Supplementary file 1 — Supplementary Material 1 [file 12890_2024_2841_MOESM1_ESM.docx]

Quality control index of lung cancer in Fujian Province from 2011 to 2020

| Year | MV(%) | DCO(%) |
| --- | --- | --- |
| 2011 | 57.29 | 0.50 |
| 2012 | 60.16 | 1.14 |
| 2013 | 61.21 | 0.27 |
| 2014 | 67.20 | 0.00 |
| 2015 | 67.75 | 0.02 |
| 2016 | 70.92 | 0.18 |
| 2017 | 69.50 | 0.30 |
| 2018 | 73.54 | 0.60 |
| 2019 | 78.78 | 0.19 |
| 2020 | 82.90 | 0.06 |

MV: morphologically verified; DCO: death certificate only.
